# Feeding and swallowing difficulties in previously hospitalised premature infants in the neonatal intensive care unit: Perspectives from South African caregivers

**DOI:** 10.4102/sajcd.v72i2.1119

**Published:** 2025-11-25

**Authors:** Cynthia Sawasawa, Lavanya Naidoo, Salma Sheik Hoosen

**Affiliations:** 1 Department of Speech Pathology and Audiology, Faculty of Humanities, University of the Witwatersrand, Johannesburg, South Africa

**Keywords:** premature infants, NICU, attachment and bonding, South Africa, emotional distress, information giving practices, feeding and swallowing difficulties

## Abstract

**Background:**

Premature birth is associated with many medical conditions including feeding and swallowing difficulties. Despite the existing knowledge on caregivers’ experiences, there is a dearth of literature that has explored experiences of caregivers whose infants have feeding difficulties in the neonatal intensive care unit (NICU) in South Africa.

**Objectives:**

This study explored experiences of caregivers of premature infants diagnosed with feeding difficulties in the NICU within the South African context.

**Method:**

This study was conducted using an explorative qualitative design. Data were collected using an anonymous qualitative survey distributed through caregiver social media support groups. Purposive sampling was used to recruit 9 participants. Data were analysed using inductive thematic analysis.

**Results:**

Three main themes emerged, namely: (1) Emotional distress associated with infants’ feeding and swallowing difficulties and prematurity, (2) infants’ inability to feed orally and NICU restrictions: barriers to caregiver-infant attachment and bonding, and (3) caregiver informational needs regarding oral feeding and general child care.

**Conclusion:**

This study indicates the need for holistic family-centred healthcare practices within the NICU. Therefore, in addition to providing oral-sensorimotor intervention to infants with feeding and/or swallowing difficulties, speech-language therapists (SLTs) need to ensure that caregiver needs and concerns related to their child are addressed. Moreover, SLT interventions need to consider caregiver-infant attachment and bonding strategies in the absence of oral feeding.

**Contribution:**

This study highlights the need for family-centred care practices within the NICU, which considers informational needs, inclusion in oral-sensorimotor intervention, and the psychosocial needs of caregivers.

## Introduction

Prematurity is a global concern with increased incidence rates reported in the Global South (Buys & Gerber, 2021; Kamran et al., 2023). Premature infants are thus a vulnerable population group given the multitude of medical conditions that are frequently associated with prematurity, such as respiratory distress syndrome and intraventricular haemorrhages (Undela et al., 2019). In addition, the ability to feed and swallow is often impaired in premature infants, which can necessitate tube feeding alternatives to ensure adequate nutritional intake and weight gain (Kamity et al., 2021). It is estimated that between 40% and 70% of premature infants present with immature feeding skills that impair their ability to feed orally, thus necessitating the need for tube feeding (Capilouto et al., 2019; Pineda et al., 2018; Wahyuni et al., 2022). Most infants experience challenges transitioning from tube feeds to full oral feeds, which further prolongs their stay in the neonatal intensive care unit (NICU) while increasing their risk for hospital acquired infections (Hendy et al., 2024; Kamran et al., 2023). The medical complications of a preterm birth can alter feeding experiences, thereby delaying the initiation and progression to full oral feeds (Osman, 2019).

Premature infants present with feeding and swallowing difficulties, which may occur at all phases of swallowing (Kamity et al., 2021). These difficulties may present as an absent or weak suck, poor oral motor skills, poor suck-swallow-breathe (SSB) coordination, nasal regurgitation and aspiration (Ostadi et al., 2021). The prolonged use of tube feeding increases the possibility of oral sensory aversion, further impacting early feeding development (Bingham, 2009). In addition, the use of mechanical ventilation has been found to delay the development and maturation of oral motor skills (Dewi et al., 2024). Therefore, invasive medical procedures, excessive handling and neurological immaturity decrease opportunities for oral feeding and positive feeding experiences (Dewi et al., 2024). This article will add to existing knowledge by describing how factors related to prematurity, caregivers, and the practices within the NICU environment impact caregivers’ experiences in the South African context.

### Experiences of caregivers

Feeding and swallowing difficulties in preterm infants can have a significant impact on caregivers, particularly mothers (Vizzari et al., 2023). In their study, that explored cue-based co-regulated feeding, Shaker (2013) found that mothers in the NICU experienced stress and anxiety because they blamed themselves for their infants’ feeding difficulties. A decline in the interactions between mothers in Iran and their infants during tube feeding negatively impacted caregiver-infant bonding and attachment (Kamran et al., 2023). However, these mothers reportedly experienced less stress, as their child’s tube feeds ensured adequate nutrition and rapid weight gain (Kamran et al., 2023). Similarly, mothers in Brazil reported that although they experienced the initial disappointment of not being able to breastfeed their infants, they were satisfied with the observable benefits of tube feeding (Banhara et al., 2020). In contrast, Buys and Gerber (2021) found that mothers in a hospital in South Africa reported negative experiences with tube feeding because of the observable discomfort of the infants.

The infants’ transition from tube feeding to oral feeding is also stressful for mothers because their infants’ feeding success is one of the determining factors for discharge (Van Schalkwyk & Gerber, 2021). Mothers have also described challenges with initiating breastfeeding after the transition from tube feeding because of factors such as difficulty latching, weak suck and infant refusal to breastfeed (Madiba & Sengane, 2021). The studies reviewed offer valuable insights into mothers’ experiences during tube feeding and the transition to oral feeding, as well as the impact of feeding and swallowing difficulties on attachment and bonding. However, these studies are limited as they do not address how caregivers are actively involved in oral-sensorimotor interventions. Furthermore, they provide little to no discussion on how attachment and bonding might be supported in the absence of oral feeding.

### The neonatal intensive care environment and the impact on feeding

Premature birth and the NICU admission are often associated with increased parental stress owing to factors such as increased concerns about the infant’s health and severity of their illness, altered and limited parenting roles, noise from equipment within the NICU and disruptions to caregiver-infant bonding (Hagen et al., 2019; Hendy et al., 2024). Mothers in Sweden described challenges when attempting breastfeeding as they had not received practical support on how to handle their infant while all the ‘wires’ and ‘tubes’ were attached (Ericson & Palmér, 2019). While in South Africa, caregivers found the NICU environment stressful because of the strict feeding routines, their inability to identify hunger cues and their limited understanding of their infants’ feeding difficulties (Van Schalkwyk & Gerber, 2021). Hence, traditional parenting roles are often altered within the NICU because of the barriers that exist within this environment (Nyaloko et al., 2024; Pineda et al., 2018). Therefore, many parents are unable to perform typical caregiving tasks such as feeding, holding and touching their infants, thus negatively impacting their level of interaction with their premature infants (Nyaloko et al., 2024).

While research has been conducted on the clinical management of feeding difficulties in preterm infants globally, it is important to continuously explore the experiences of caregivers in low-resourced contexts such as South Africa to ensure that speech-language therapists (SLTs) provide contextually and culturally relevant services that align with the needs of the caregivers and their premature infants. In addition, the social, cultural and environmental contexts, unique to the perspectives of South African caregivers, remain under-represented in available literature sources. This study thus aimed to explore caregivers’ experiences related to their infant with feeding difficulties in the NICU and post-discharge. It also examined the nature of the training initiatives and support provided to caregivers who received services in either public or private healthcare facilities across the country. This study further explored the factors within the NICU that impacted caregiver-infant attachment.

## Research methods and design

This study was conducted using an explorative qualitative research design. This research design enabled the collection of data related to subjective lived experiences from a heterogeneous group of caregivers who shared the common experience of having a premature infant who was admitted to the NICU and subsequently had feeding difficulties (Creswell & Poth, 2018). Purposive sampling was used to collect data as it enabled the selection of participants who met a specified criterion (Campbell et al., 2020). Specific inclusion criteria included: (1) caregivers over the age of 18 years, (2) caregivers of infants who had been previously admitted to the NICU and were 6 months post-discharge, (3) infants who have received care from either a public or private healthcare facility in South Africa, (4) caregivers who were involved in the caring of the infant while admitted to the NICU and post-discharge, (5) caregivers with access to an electronic device for the completion of the online survey, and (6) caregivers of infants who presented with feeding difficulties. Table 1 describes the participants who were included in this study.

**TABLE 1:** Participant characteristics.

Participant number	Province	Geographical setting	Relationship with the child	Caregiver age range (years)	Premature infants’ age at birth (weeks)	NICU location (public or private hospital)	Length of stay in NICU	Infant feeding difficulty
P001	Gauteng	Urban	Mother	25–29	26	Private hospital	8 months	Unable to complete feeds; poor SSB coordination; oral aversion
P002	Gauteng	Urban	Mother	35–39	33	Private hospital	3 weeks	Difficulty completing feeds
P003	Gauteng	Urban	Mother	30–34	36	Private hospital	10 days	Difficulty completing feeds
P004	Gauteng	Urban	Mother	> 40	35	Private hospital	3 weeks	Difficulty completing feeds
P005	Gauteng	Township	Mother	30–34	24	Public hospital	2 months	Weak or no suck
P006	KwaZulu-Natal	Urban	Mother	21–24	26	Public hospital	2 months	Difficulty completing feeds
P007	Gauteng	Township	Mother	> 40	26	Public hospital	2 months	Weak or no suck
P008	Western Cape	Urban	Mother	21–24	26	Private hospital	1 month	Weak or no suck
P009	Limpopo	Township	Mother	21–24	36	Public hospital	3 weeks	Difficulty completing feeds

NICU, neonatal intensive care unit; SSB, suck-swallow-breathe.

### Participants’ description

A total of 9 participants were included in the study. The sample size was selected because of the explorative research design and because small sample sizes are recommended for qualitative studies (Vasileiou et al., 2018). The participants’ ages were distributed across the following ranges: 21–24 years (*n* = 3), 25–29 years (*n* = 1), 30–34 years (*n* = 2), 35–39 years (*n* = 1), over 40 years (*n* = 2).All the participants were mothers of the children. The majority of participants accessed private health care services, while four participants accessed public health care services. The majority of the participants (*n* = 6) resided in urban areas and three participants lived in townships. The gestational ages of the infants at birth ranged from 24 weeks to 36 weeks, with a mean of 26 weeks. The premature infants’ length of stay in the NICU ranged between 10 days and 2 months, with a single outlier whose stay lasted 8 months. The common feeding difficulties reported by caregivers included: difficulty completing feeding, poor SSB coordination, weak or no suck and oral aversion.

### Participants’ recruitment

Participants were recruited from five private Facebook support groups for parents of premature infants. The authors requested permission to use the groups to recruit participants, which was done by sharing an information poster that also included a link to the survey. Notably, the groups were not composed exclusively of South African parents, given that some were founded by individuals outside of South Africa. To ensure that only South African participants were included in the study, respondents were required to indicate the province in which they reside. The survey was posted by Salma Sheik Hoosen on the different groups weekly for a total of 8 weeks. The instrument used for the data collection was an online, anonymous qualitative survey, which was created using a Google Form (see Online Appendix 1). Participants accessed the survey using the link posted on the various Facebook groups. Qualitative surveys offer a ‘wide-angle lens’ on the subject of interest that has the potential to capture a diversity of viewpoints, experiences or perceptions (Creswell & Poth, 2018). Furthermore, the surveys allowed for the participation of individuals from a variety of backgrounds, social contexts and locations where their lived experiences may differ, which aligned with the study’s research design.

### Data collection instrument

The survey was self-developed by the Salma Sheik Hoosen and Cynthia Sawasawa using literature, which was undergirded by the family-centred care (FCC) approach (McCarthy & Guerin, 2022). The survey consisted of closed- and open-ended questions (see Online Appendix 1). The open-ended questions explored the following: the role and level of the caregivers’ involvement during feeding while exploring their perceived barriers and facilitators, the information that caregivers received about their infants’ feeding difficulties, the barriers and facilitators that caregivers experienced within the NICU environment, the perceived impact of the feeding difficulties on caregiver bonding and attachment, the perceived impact of the NICU admission and subsequent feeding difficulties on the rest of the family, the emotional impact of having a premature infant with feeding difficulties, and the challenges that caregivers faced after discharge.

### Data analysis

Inductive thematic analysis was used to analyse the data (Braun & Clark, 2006). The process of conducting thematic analysis was conducted using the six steps described by Braun and Clarke (2006), namely: familiarisation, coding, generating themes, reviewing themes, defining themes and labelling themes, and writing up. Data analysis began with the authors familiarising themselves with the data, which involved the reading and re-reading of the data. The initial codes were created by Cynthia Sawasawa and then reviewed by Lavanya Naidoo and Salma Sheik Hoosen. Once the codes were established, themes were then generated using the patterns identified within the data and the research aims and objectives. These themes were reviewed and further refined, a process that was facilitated by frequent discussions between the authors. Lastly, themes were labelled ensuring that they accurately capture the essence of the participants’ experiences and overall findings.

### Ethical considerations

Ethical approval for the study was granted by the Human Research Committee (Non-Medical) at the University of the Witwatersrand (approval number: STA_2023_19). The introductory page of the online survey provided information on the purpose of the survey, consent, voluntary participation and the option to withdraw at any time, confidentiality and anonymity and data processing. All of which the participants had to agree to do before proceeding with the survey. Participants’ anonymity was maintained as no personal identifiable information was collected during the survey, and alphanumeric codes were assigned to participants during data analysis. Confidentiality was maintained by ensuring that no personal identifying information was collected. In addition, the participants’ internet protocol (IP) addresses were not collected. All data were stored on a password-protected cloud-based drive, which was only accessible to the authors.

### Rigour and trustworthiness

Rigour and trustworthiness were ensured by adhering to the following strategies: credibility, dependability and transferability (Forero et al., 2018; Johnson et al., 2020). Credibility was established through peer debriefing among the authors, which limited potential bias during the analysis and interpretation of the data (Forero et al., 2018). Dependability was achieved by providing rich descriptions of the methodological procedures that were followed in this study (Johnson et al., 2020). Transferability was achieved by providing detailed information about the participants’ socio-demographic characteristics, which would assist in determining the generalisability of the findings (Johnson et al., 2020).

## Results

Three themes emerged in this study, namely: (1) Emotional distress associated with infants’ feeding and swallowing difficulties and prematurity, (2) infants’ inability to feed orally and NICU restrictions: Barriers to caregiver-infant attachment and bonding, and (3) caregiver informational needs regarding oral feeding and general child care. The findings presented here also indicate that the experiences of participants were similar, irrespective of whether they accessed public or private healthcare services. Additionally, the infant’s length of stay in the NICU did not appear to be a factor that influenced the participants’ experiences because they all reported similar experiences regardless of their hospital duration.

### Theme 1: Emotional distress associated with infants’ feeding and swallowing difficulties and prematurity

Most participants expressed emotional distress characterised by feelings of worry and fear when they were informed about their infants’ feeding difficulties. These feeding difficulties were characterised as weak or no suck and difficulty completing feeds (which could be related to poor endurance)with some infants presenting with difficulties related to the uncoordinated SSB rhythm. The feeding difficulties resulted in increased concerns about delayed discharge and poor weight gain:

‘I was very worried as I kept wondering what would sustain the baby if they can’t eat.’ (P005, Mother, Public Hospital)

‘I had concerns around it [*feeding difficulties*] delaying his release from hospital. I was comfortable knowing that he was tube fed and that he would get his nutrition from that. However, the doctor would only release him if he was drinking a certain amount in a certain time.’ (P004, Mother, Private Hospital)

A few participants expressed feelings of helplessness and self-blame because they felt as though they had failed in their role as mothers, since they could not do anything about the feeding difficulties that their infants presented with:

‘I was distraught. This is [*the*] first baby I have. The baby was a new life form that has been put in my care, but I couldn’t do anything to help it thrive the way I was hoping too. I felt so helpless and lost. I began thinking that I had just started my role as a mother and had already failed that role.’ (P008, Mother, Private Hospital)

‘I was worried and scared. I didn’t know what that meant and how my baby was going to develop after knowing that. I wasn’t sure how I was meant to proceed.’ (P006, Mother, Public Hospital)

One mother reported that she lost her maternal identity and felt as though she had failed in her mothering role because she was unable to feed her infant orally through breastfeeding and provide the overall care that her infant needed:

‘My baby was very unresponsive to the feeding. Sometimes I would worry that my baby is not getting the proper food and nutrition. I felt like I wasn’t able to be a mother without breastfeeding the baby. A mother’s main role is to provide and care however I had failed in both areas.’ (P008, Mother, Private Hospital)

The participants’ emotional distress was further exacerbated by the fact that they had given birth to a premature infant. Some participants reported that they were anxious about how the prematurity would impact developmental milestones, while others did not understand what to expect:

‘I was confused. I honestly did not know what it meant to have a premature baby. I was informed that by two years the baby should be achieving milestones according to regular kids that aren’t born premature. I just felt like I will need to take better care of my baby because of this.’ (P008, Mother, Private Hospital)

‘I was quite nervous and sad. I did not know what to expect.’ (P009, Mother, Public Hospital)

There were a few participants who felt guilty because they thought they had done something during pregnancy to cause the prematurity, often internalising their infant’s early delivery as a personal failure casting doubt on their thoughts of future pregnancies:

‘I was disappointed and traumatised. Felt like I had done something wrong in my pregnancy to cause the prem [*preterm*] birth.’ (P001, Mother, Private Hospital)

‘I was scared and thought that I did something wrong. I felt like it was my fault that this had happened, and it made me scared of ever wanting another child.’ (P006, Mother, Public Hospital)

Participants indicated that their extended family members experienced emotional distress because of the prematurity and fear of the infant dying:

‘My family was very anxious especially because at some point we were also told to expect and prepare for the worst as [*the*] baby was born way too soon.’ (P005, Mother, Public Hospital)

‘We were all very anxious and many of us [*the participant and her family*] could not get much rest. There was a constant worry for the baby even sleep didn’t feel like proper rest because our minds were still racing with thoughts of whether the baby will live to see another day or whether the baby was okay.’ (P006, Mother, Public Hospital)

### Theme 2: Infants’ inability to feed orally and neonatal intensive care unit restrictions: Barriers to caregiver-infant attachment and bonding

Most of the participants described feeling distressed because of the emotional and physical barriers that prevented their bonding with their child. This they referenced in particular as related to their restricted physical contact and inability to breastfeed their child during infancy:

‘I wished I was able to breastfeed as I knew that breastfeeding was a way of bonding with the baby but unfortunately I could not do that.’ (P005, Mother, Public Hospital)

‘I wasn’t allowed close to the baby for the first two or three weeks. And she also had a tube in her nose and this gave me anxiety. I was scared of touching it or moving it.’ (P006, Mother, Public Hospital)

The restricted physical contact between participants and the premature infants resulted in a shift of caregiving responsibilities from the participants to the nurses:

‘Initially I was unable to hold her and had to watch the nurses feeding her.’ (P003, Mother, Private Hospital)

A few participants indicated that NICU hospital restrictions also limited their opportunities for bonding, with one participant describing that she only had the opportunity to bond with her infant once he was discharged:

‘I did not bond with the baby until he was discharged. Skin to skin was difficult because of the tubes and wires. I did not have a significant amount of time to bond.’ (P004, Mother, Private Hospital)

While another participant indicated that her spouse was unable to bond with their premature infant because he was not allowed in the NICU:

‘My husband was not allowed in the NICU which also burdened him because he had to hear how his baby was doing from word of mouth. And it also affected how well he could bond with the baby.’ (P008, Mother, Private Hospital)

### Theme 3: Caregiver informational needs regarding oral feeding and general child care

Most participants indicated that they were satisfied with the training they had received when their infants were ready to begin oral feeds because the training included practical aspects and was provided in great detail:

‘I liked that they had tried to guide me through it instead of giving me the instructions and leaving me to my own devices. They had stood with me and guided me through procedures like stimulating a suck. And how to feed and care for the baby.’ (P006, Mother, Public Hospital)

‘Everything was explained clearly so I could feed my baby with no problems.’ (P007, Mother, Public Hospital)

There were, however, participants who reported that they still had unmet information needs regarding their infants’ feeding difficulties and prognosis:

‘I would have liked to know that tube dependence is a real thing. I didn’t know about this until it was too late. I understand that he would have gotten it anyway because he was born so early and needed the tube for a long time. But it would have been nice to know about.’ (P001, Mother, Private Hospital)

‘I actually just wanted to really know why she had feeding difficulties. No one really explained exactly what was wrong to me. I was just told oh this is the issue.’ (P006, Mother, Public Hospital)

Participants’ informational needs continued post-discharge, which implied that caregivers did not receive follow-up care from SLTs once their infants were discharged from the NICU. Some participants relied on social media platforms such as YouTube, books and the knowledge of their family members:

‘Self-taught by following YouTube videos. Finding the correct position that works for us, ways to position baby’s mouth to get a proper latch and using an app to track and time the feeds.’ (P004, Mother, Private Hospital)

‘My grandmother was a nurse and she had helped a lot with explaining how the baby needs to suck and how I need to find a speech therapist to guide me.’ (P006, Mother, Public Hospital)

## Discussion

The findings of this study indicate that caregivers of premature infants with reported feeding difficulties experienced increased emotional distress because of their child’s prematurity diagnosis, secondary feeding difficulties, self-blame regarding the premature birth, and uncertainty about their child’s prognosis. The findings also highlighted that when caregivers are excluded from participating in routine caregiving tasks, such as feeding and bathing, and have limited opportunities to bond and attach with their infants, they have a reduced sense of their maternal role. Lastly, the findings of this study revealed different perspectives regarding the amount of information and training given to caregivers related to their children’s feeding difficulties.

The perceived feeding challenges described by the participants in this study, namely, the child’s weak or absent suck, uncoordinated SSB patterns, poor endurance and a reliance on tube feeding, are all consistent with literature and are well-documented (Da Costa et al., 2019; Osman, 2019; Ostadi et al., 2021; Rachmawati et al., 2024; Wahyuni et al., 2022). The caregivers’ concerns about poor weight gain and delayed discharge are also consistent with findings from earlier research that emphasises feeding readiness as a crucial factor in NICU length of stay and infant health outcomes (El-Kassas et al., 2024; Lau, 2018; Lyne et al., 2022; Madiba & Sengane, 2021). The significance of early and focused interventions to support feeding in this population, as well as the necessity of caregiver education to increase knowledge and lessen anxiety regarding feeding difficulties in premature infants, are highlighted by the congruence of caregiver-reported experiences with clinical research (El-Kassas et al., 2024; Lau, 2018; Lyne et al., 2022).

A particular theme that resonated with many participants was the emotional burden of their child’s feeding and swallowing difficulties, compounded by their child’s prematurity. These challenges were emphasised alongside their feelings of stress and fear concerning their child’s long-term developmental outcomes and immediate nutritional risks. Caregivers consistently indicated feelings of worry, internalised helplessness and guilt. This finding aligns with the research by Lean et al. (2018) who note that the experience of giving birth to a premature infant who is subsequently admitted to the NICU is often associated with various negative emotions, such as those described by participants in this study. Similarly, the study by Baía et al. (2016) found that emotional distress was common among mothers of premature infants in Portugal. Mothers in South Africa reported similar experiences, as indicated by Nyaloko et al. (2024) who found that mothers experienced heightened anxiety, stress and exhaustion and a constant state of fear. In this study, emotional distress was reported by most participants irrespective of the healthcare setting in which their premature infant received medical care, that is, private versus public healthcare settings. This finding suggests that the emotions caregivers experience within the NICU environment are not specific to any particular healthcare context and transcend the caregiver’s socio-economic context. Moreover, our findings and corroborating literature highlight the need to incorporate more FCC practices that consider both the child and the caregiver. Thus, psychosocial support should be provided to caregivers while their children are admitted to the NICU, as many factors, such as feeding and swallowing difficulties, affect their emotional well-being.

The perceived loss of the caregiver role following their infants’ admission to the NICU was viewed as a significant contributor to the caregivers’ emotional distress. These findings were corroborated by Baía et al. (2016) who reported that mothers and fathers reported increased stress when their parental role was changed, as they were not considered the primary caregivers within the NICU environment. Similarly, Samane et al. (2022) reported that the premature infant’s admission to the NICU leaves parents feeling powerless because of their limited involvement in the care of the infant. These findings further underscore the need for active involvement of caregivers in care tasks within the NICU, which would facilitate increased caregiver-infant attachment (Pineda et al., 2018).

Participants reported that they had difficulty establishing bonding with their infants, especially in instances where they were unable to breastfeed because of their infants’ feeding difficulties, medical status and NICU regulations. These factors essentially limited caregiver opportunities to develop physical and emotional connections with their infant. This finding echoes the results from other similar studies; for example, Kamran et al. (2023) found that mothers in Iran with infants who were tube-fed interacted less with their infants as compared to when their infants were fed orally. Interestingly, perceived difficulty with bonding was described by mothers in South Africa who were, in fact, breastfeeding and/or cup feeding their infants because feeding was task-oriented and attached to the goal of discharge instead of being an opportunity for mothers to bond with their infants (Van Schalkwyk & Gerber, 2021). These findings suggest the need for SLTs to educate caregivers on other strategies, such as early language stimulation techniques that can facilitate bonding and attachments. Moreover, caregivers should also be encouraged to perform kangaroo-mother care (KMC) while tube feeding their infants, which can be performed through the advocacy of SLTs. According to Sjömar et al. (2023), KMC facilitates caregiver-infant attachment, empowers mothers and reduces the risk of postpartum depression. Additionally, once the infants’ feeding skills have developed and the transition to oral feeds begins, mothers should be given the opportunity to breastfeed their infants even if initially it is for short intervals. A process that can be facilitated by SLTs in collaboration with dieticians and lactation consultants.

While some participants reported receiving adequate information and training regarding how to support their infant while feeding, others reported gaps in their understanding of their child’s specific feeding challenges as related to their prematurity and their prognosis. The lack of information resulted in the participants feeling unprepared and compelled to source information independently post-discharge. These results are similar to those reported by Ericson and Palmér (2019), where some Swedish mothers were satisfied with the training and information they received when breastfeeding was initiated, because they were provided with individualised training that was not only practical but was also provided in a caring and supportive manner. In the same study, some mothers indicated that they received inadequate support because they received conflicting information from healthcare professionals, while others felt unsupported because they were not encouraged to breastfeed (Ericson & Palmér, 2019). In contrast, Van Schalkwyk and Gerber (2021) emphasised how mothers improved their feeding skills through the direct instructions given by healthcare professionals, in addition to observing how the healthcare professionals fed the infants. The caregiver’s dependency on external sources such as YouTube and other social media platforms further reflects the need for continuous and personalised caregiver education. Here, SLTs are encouraged to frequently enquire from caregivers about their informational needs regarding their infants’ feeding and swallowing. This process will ensure that information giving is tailored to the needs and concerns of the caregivers and not limited to information that the clinician chooses to share. Additionally, as highlighted by the participants in this study, SLTs should also provide hands-on practical information where caregivers are shown how to implement treatment strategies, positioning during feeding and how to recognise feeding and swallowing difficulties such as aspiration. Moreover, the post-discharge information needs described by participants indicate that SLTs should ensure that premature infants who previously presented with feeding and/or swallowing difficulties while admitted to the NICU, are followed up post-discharge.

### Implications

#### Clinical

The findings from this study emphasise the pervasive emotional and psychological burden experienced by caregivers of premature infants, who present with feeding difficulties. There is, therefore, a need for SLTs and other allied healthcare professionals to advocate for increased caregiver psychosocial support in both private and public healthcare facilities. The disproportionate patient-provider ratio within the public sector may make it challenging for caregivers to receive individual psychosocial support. Therefore, SLTs in conjunction with social workers and/or psychologists can facilitate the development of support groups for caregivers with premature infants diagnosed with feeding and/or swallowing difficulties who are currently admitted to the NICU. The groups would eventually be caregiver-led, which further empowers caregivers. To improve caregiver-infant attachment in the absence of oral feeding, SLTs should encourage the implementation of KMC during tube feeding. The implementation of KMC can only be achieved through collaborative efforts between SLTs and nursing staff who typically spend more time with the infant and the caregiver. Furthermore, nurses will be able to provide practical guidance on how to conduct KMC on premature infants attached to various monitoring cables, supplementary oxygen and feeding tubes, thus preventing dislodgment. Lastly, there is a need for improved collaboration between healthcare professionals within the NICU to facilitate integrated implementation of FCC principles, which will in turn ensure that caregivers receive the support, information and care that they need across the continuum of care.

#### Undergraduate training

The practical implementation of the FCC model should be emphasised in the undergraduate training curriculum for SLTs. This emphasis will ensure that students are provided with the knowledge and practical skills needed to provide services that are contextually relevant and cognisant of the role of the caregiver when treating infants. Moreover, the paediatric dysphagia module taught to SLTs should include topics such as attachment and bonding, specifically how SLTs can facilitate the caregiver-infant attachment in tube-fed infants receiving oral-sensorimotor intervention.

### Limitations of the study and recommendations for future research

While the study’s findings may be relevant to public and private sector caregivers, we acknowledge that the findings may not be generalisable to all healthcare contexts, for example, those living in rural areas. The findings did not include information about the socio-economic status of the participants, nor their education level, which has been suggested by previous literature to impact the experiences of mothers within the NICU. It is recommended that future research focus on a larger, more representative sample, which will ensure the generalisability of the findings in various contexts and healthcare settings in South Africa. In addition, data should be collected from a heterogeneous, linguistically and culturally diverse sample. The study’s inclusion criteria were limited to individuals who had access to internet-enabled devices that allowed them to access the survey. Furthermore, the study only included participants who are literate in English. The use of the qualitative survey as opposed to individual interviews limited the authors’ opportunities to probe participants for further information that may have improved the richness of the data. It is therefore recommended that future research in this population be conducted using individual semi-structured interviews.

## Conclusion

This study highlights the need for holistic healthcare practices that take into consideration the needs of premature infants and their caregivers. Therefore, in addition to providing oral-sensorimotor interventions to infants with feeding and/or swallowing difficulties, SLTs need to ensure that caregivers’ needs and concerns related to their child are addressed. In addition, the intervention strategies implemented by SLTs should include aspects of attachment and bonding, KMC, and strategies for increased caregiver participation, especially for infants who are tube-fed. Lastly, there is a need for more collaboration among the healthcare professionals treating premature infants admitted to the NICU so as to ensure that the holistic needs of the infant and caregiver are addressed during hospitalisation and post-discharge.
